# Discovery of Post-Translational Modifications in *Emiliania huxleyi*

**DOI:** 10.3390/molecules26072027

**Published:** 2021-04-02

**Authors:** Van-An Duong, Onyou Nam, EonSeon Jin, Jong-Moon Park, Hookeun Lee

**Affiliations:** 1 College of Pharmacy, Gachon University, Incheon 21936, Korea; anduong@gachon.ac.kr; 2 Department of Life Science, Research Institute for Natural Sciences, Hanyang University, Seoul 04763, Korea; oynam@hanyang.ac.kr (O.N.); esjin@hanyang.ac.kr (E.J.)

**Keywords:** post-translational modifications, *Emiliania huxleyi*, mass spectrometry, three-dimensional liquid chromatography, proteomics

## Abstract

*Emiliania huxleyi* is a cosmopolitan coccolithophore that plays an essential role in global carbon and sulfur cycling, and contributes to marine cloud formation and climate regulation. Previously, the proteomic profile of *Emiliania huxleyi* was investigated using a three-dimensional separation strategy combined with liquid chromatography-tandem mass spectrometry (LC-MS/MS). The current study reuses the MS/MS spectra obtained, for the global discovery of post-translational modifications (PTMs) in this species without specific enrichment methods. Twenty-five different PTM types were examined using Trans-Proteomic Pipeline (Comet and PeptideProphet). Overall, 13,483 PTMs were identified in 7421 proteins. Methylation was the most frequent PTM with more than 2800 modified sites, and lysine was the most frequently modified amino acid with more than 4000 PTMs. The number of proteins identified increased by 22.5% to 18,780 after performing the PTM search. Compared to intact peptides, the intensities of some modified peptides were superior or equivalent. The intensities of some proteins increased dramatically after the PTM search. Gene ontology analysis revealed that protein persulfidation was related to photosynthesis in *Emiliania huxleyi*. Additionally, various membrane proteins were found to be phosphorylated. Thus, our global PTM discovery platform provides an overview of PTMs in the species and prompts further studies to uncover their biological functions. The combination of a three-dimensional separation method with global PTM search is a promising approach for the identification and discovery of PTMs in other species.

## 1. Introduction

Post-translational modifications (PTMs) of proteins are proteolytic cleavages or covalent addition of modifying groups to amino acids after protein biosynthesis. They play essential roles in regulating protein function, stability, complex formation, localization, and protein-protein interactions [1,2]. To date, approximately 1500 different PTMs have been reported and are included in the Unimod database. Bottom-up proteomics approaches have been used for PTM discovery using liquid chromatography-tandem mass spectrometry (LC-MS/MS) [3]. The analysis of PTMs by MS depends on the overall abundance of the modified peptides, complexity of biological samples, and stability of the PTMs during MS and MS/MS analysis [4]. The detection of PTMs by MS mainly relies on the change in the masses of peptides bearing PTMs, which results from chemical modifications occurring in their amino acids. MS/MS can unequivocally assign a given modification to a given site by observing the mass shift in the precursor ion and the fragment ions carrying the modifications [5]. MS/MS data have been used for global PTM identification at the proteome level with the help of specific search tools, for example, Sequest, X!Tandem, InsPecT, MaxQuant, and MODa [6,7]. Additionally, Comet incorporated in Trans-proteomic Pipeline (TPP) can also be used for identifying PTMs from MS/MS spectra [8]. Different PTMs can be identified in the TPP by altering the search parameters (variable modifications) [9]. Several PTM enrichment techniques have been applied to increase the number of PTMs detected, such as immunoaffinity chromatography [10], immobilized metal ion-affinity chromatography [11], titanium dioxide [12] for phosphorylation, peptide-N-glycosidase F and lectin for glycosylation [13,14], and immunoaffinity purification with anti-acetyl-lysine antibodies for acetylation [15]. In addition, sample fractionation can be carried out to reduce the sample complexity and increase the number of peptides and PTMs identified [16]. Sample fractionation for global PTM identification in *Synechococcus* was previously reported using two-dimensional LC, which revealed nearly 12,000 sites of 23 different PTMs in 6704 unique peptides and 2230 proteins [6].

*Emiliania huxleyi* is a cosmopolitan coccolithophore that has attracted researchers from various fields, including medicine, material science, biogeography, geology, ecophysiology, and paleoclimatology [17]. It plays an essential role in global carbon and sulfur cycling, and contributes to marine cloud formation and climate regulation [18,19]. Like other coccolithophores, *Emiliania huxleyi* can fix inorganic carbon into biomineralized and photosynthetic products. It can also produce CaCO_3_ coccolith plates surrounding the cell, which contribute to the formation of chalk and limestone sediments [18]. Various studies have been conducted on *Emiliania huxleyi* due to its importance in global biogeochemistry [20,21,22]. The genome of this species has been sequenced [23]; however, only a few studies have performed proteomic analyses of *Emiliania huxleyi* [24,25,26]. We recently developed three-dimensional liquid chromatography (3D-LC) coupled with MS/MS for in-depth proteomic profiling of this species. The proteome digest of *Emiliania huxleyi* was fractionated using strong cation exchange and high pH reversed-phase LC, which yielded 70 fractions for LC-MS/MS analysis. The platform identified more than 84,000 peptides and 15,000 protein groups (including single hits) [27]. The same proteomic datasets can be used to extract further information, particularly PTMs [6,28], which are unknown in *Emiliania huxleyi.* Thus, in this study, we repurposed a dataset from a previous study [27] for the global discovery of PTMs in *Emiliania huxleyi*. We examined 25 different PTMs in this species using Comet search without specific enrichment methods and investigated the roles of some PTM types.

## 2. Results and Discussion

### 2.1. Global PTM Discovery from MS/MS Spectra

Identification of PTMs in this study was based on a bottom-up proteomic approach, which included protein extraction, enzymatic digestion, fractionation, and LC-MS/MS analysis. Previous studies have used enrichment methods to improve the identification of modified peptides [10,29]. Our study was aimed at the global discovery of PTMs without using any enrichment for specific PTMs. We used a dataset with in-depth proteome coverage, obtained from a previous 3D-LC separation [27], and Comet to identify 25 common types of PTMs [6]. One round of PTM search was carried out on 70 MS/MS raw files with a narrow precursor mass tolerance (10 ppm). Peptides that contained only carbamidomethylation of cysteine, oxidation of methionine, or carbamylation at the N-terminal were not considered as modified peptides. The total processing time was approximately 300 h using an LG workstation with an Intel Xeon CPU, 2.7 GHz, and a maximum of 24 threads (for 15 independent searches of 25 PTM types on 70 raw files). A previous study reported a total processing time of about 16 days (384 h) using a similar workstation, when examining 28 peptide fractions from Jurkat cell digest for 24 types of PTMs [1]. MaxQuant was also used to identify PTMs and determine the localization probability of modifications in peptides [30]. However, in our study, when using MaxQuant to identify PTMs and quantify peptides and proteins, the time required was relatively long. With five fractions (from E1 to E5) and including only lysine acetylation (Lys, K) and the methylation of glutamic acid (Glu, E)/Lys/arginine (Arg, R) to the search, the total search time was approximately 24 h.

**Figure 1:**
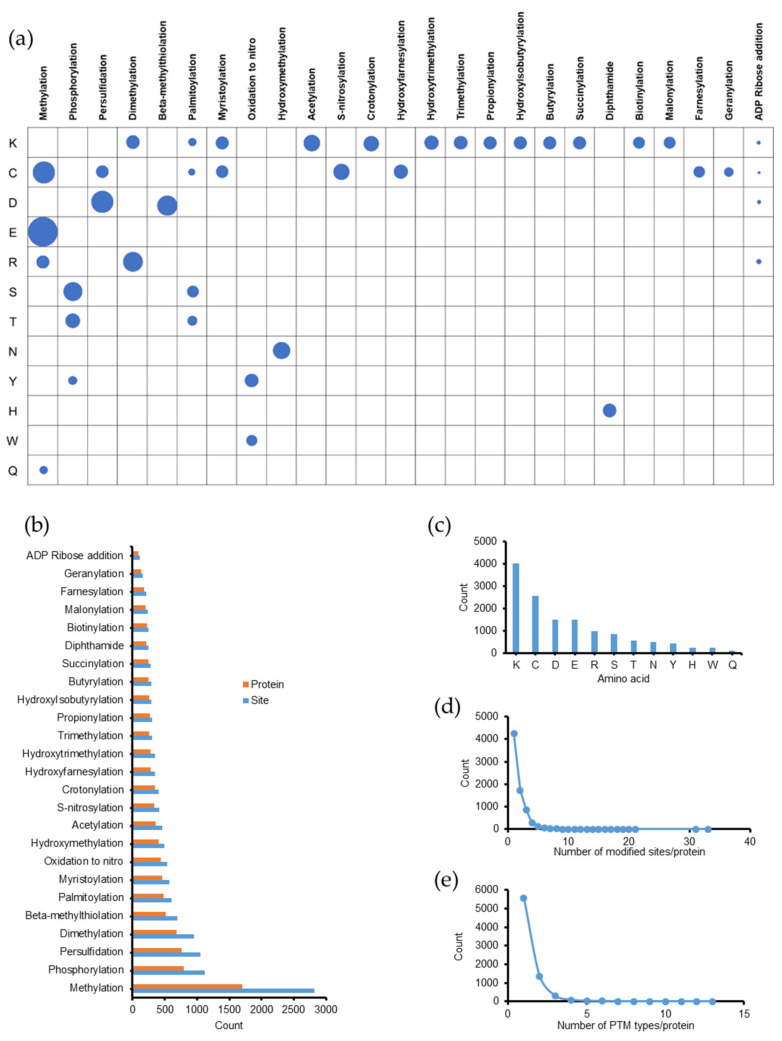
Global discovery of post-translational modifications (PTMs) in *Emiliania huxleyi* from tandem mass (MS/MS) spectra. (**a**) PTM distribution by PTM types and amino acid sites. The number of modified sites is proportional to the area of the circle. (**b**) The number of modified sites and proteins according to each type of PTM. K: lysine, C: cysteine, D: aspartic acid, E: glutamic acid, R: arginine, S: serine, T: threonine, N: asparagine, Y: tyrosine, H: histidine, W: tryptophan, and Q: glutamine. (**c**) The number of PTMs by amino acids. (**d**,**e**) The number of modified sites and PTM types in each protein.

The fractionation of samples into 70 fractions improved the proteome coverage and thereby, increased the number of PTMs identified. As a result, we found a large number of PTMs. Overall, 10,710 modified peptides were identified (Table S1) and the results are presented in Figure 1. We found 13,483 modified sites in 7421 proteins. A summary of the distribution PTMs based on their types and amino acid sites is shown in Table S2 and illustrated in Figure 1a. In addition, Figure 1b shows the number of modified sites and proteins according to each PTM type. Methylation appeared to be the most frequent PTM in *Emiliania huxleyi*, with more than 2800 modified sites on 1703 proteins, followed by phosphorylation with 1120 modified sites on 789 proteins. The numbers of PTMs identified by amino acid sites are shown in Figure 1c. Among these, Lys was the most modified (more than 4000 times), followed by cysteine (Cys, C, modified 2553 times). In this dataset, trypsin, which is a member of the serine protease family, was used for protein digestion. Its substrate-binding pocket is deep and has a negative charge at the bottom (aspartate). Therefore, only Arg and Lys, which have long positively charged side chains, are the target amino acids for trypsin cleavage. After recognizing a target amino acid in a binding pocket, trypsin cleaves the C-terminal amide bond [31]. Several PTMs were found at Lys and Arg sites, which were recognized as missed cleavage sites in the search. Hence, modified Lys and Arg were not cleaved by trypsin, as previously reported [32,33].

The number of PTM sites and types in each protein is summarized in Table S3 and illustrated in Figure 1d,e. As shown in Figure 1d, 4246 proteins (~57.2%) were modified at only one amino acid site, whereas 1727 (~23.3%) and 867 (~11.8%) proteins exhibited two and three modified sites, respectively. The remaining 581 proteins (~7.8%) contained at least four modified sites each. We found that 5575 proteins were modified by one type of PTM (Figure 1e), and among them, 1329 proteins contained at least two sites modified by the same type of PTM (Table S3). In addition, 1369 proteins were modified by at least two different PTM types. The number of different types of PTMs on one protein ranged from 1–13. Notably, one protein (Q4G3C8, ATP synthase subunit beta, chloroplastic) was modified at 18 amino acids with 13 different PTMs (one methylation, two phosphorylations, three persulfidations, one beta-methylthiolation, one myristoylation, one oxidation to nitro, one hydroxymethylation, three acetylations, one hydroxytrimethylation, one propionylation, one hydroxyisobutyrylation, one butyrylation, and one diphthamide). Another protein (R1DMT8, transketolase) was modified at 33 amino acids with 11 different PTMs (nine methylations, nine persulfidations, two beta-methylthiolations, one oxidation to nitro, three hydroxymethylations, three acetylations, one S-nitrosylation, two propionylations, one butyrylation, one succinylation, and one diphthamide).

We also manually determined 165 specific sites that were modified by at least two different PTMs (Table S4). In particular, 145, 14, and 4 sites were modified by 2, 3, and 4 different PTMs, respectively. One Lys (RK*GLSPLLRG) in Q4G3B5 (photosystem I reaction center subunit XI) was modified by five different PTMs (acetylation, crotonylation, succinylation, propionylation, and butyrylation). Another Lys (RK*TVTAMDVVYA) in R1CEA2 (histone H4) was modified by five different PTMs (trimethylation, propionylation, butyrylation, malonylation, demethylation, and acetylation). This indicates that different proteoforms of a protein can co-exist and their functions might be similar or different.

### 2.2. Effect of PTMs on the Identification of Peptides and Proteins

**Figure 2:**
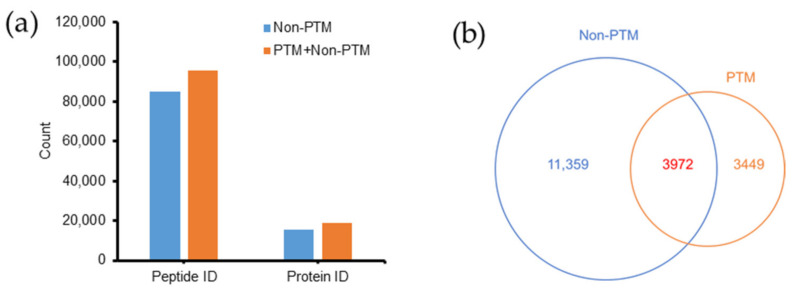
Effects of PTM search on peptide and protein identification in *Emiliania huxleyi*. (**a**) PTM search increased the number of peptides and proteins identified (IDs). (**b**) Comparison of proteins identified between non-PTM and PTM searches.

Without including PTMs, 84,753 peptides and 15,331 proteins (including single hits) were identified [27]. After incorporation of the PTM data, the number of peptides and proteins increased 12.6% to 95,463 and 22.5% to 18,780 (Figure 2a). Thus, a large number of peptides and proteins missed by conventional searches could be identified by the PTM search. A comparison of proteins identified in the non-PTM and PTM datasets is shown as a Venn diagram (Figure 2b). The PTM search contributed to the identification of 3449 new proteins that were not found in the non-PTM dataset. In addition, 3972 previously identified proteins in the non-PTM dataset were found to have PTMs.

In bottom-up proteomics, the digestion of proteins into smaller peptides results in the loss of connectivity between the different peptides of the proteins. It should be noted that the same gene, through alternative RNA splicing and PTMs, produces different proteoforms, making up a proteoform family [34]. The identification of PTMs in a bottom-up proteomic study does not allow us to determine which proteoforms are present. We found many multiply modifiable sites (Table S4), which suggested that the corresponding modified proteins co-existed in the sample. For example, five different PTMs were found on the same Lys residue (R.K*GLSPLLR.G) of photosystem I reaction center subunit XI (Q4G3B5), including acetylation, crotonylation, succinylation, propionylation, and butyrylation. Thus, five different proteins, each containing one of these PTMs, are present in the cell simultaneously. However, in other cases, when proteins contain multiple modified sites, it becomes difficult to determine how many modified proteins exist. For example, protein photosystem II protein D1 (Q4G3F2) contained four modified peptides as follows:

Peptide 1: R.n[4 4.01]SN[144.05]LGMEVMHER.N (hydroxylmethylation on Asn).
Peptide 2: R.n[44.01]E[143.06]TTENESANYGYK.F (methylation on Glu).
Peptide 3: R.n[44.01]E[143.06]WELSYR.L (methylation on Glu).
Peptide 4: K.FGQEEETY[208.05]NIVAAHGYFGR.L (oxidation on Tyr).

This protein could exist in different modified forms (proteoforms): four proteins with only one modified peptide, six proteins with two of the four modified peptides, four proteins with three of the four modified peptides, and one protein with all four modified peptides. This is one of the limitations of bottom-up proteomics [35]; however, some advances in top-down proteomics could help to overcome this. The combination of bottom-up, top-down [36], and middle-down [37] approaches may allow a comprehensive study of complex PTM patterns.

### 2.3. Effects of PTMs on Quantitative Analysis

**Figure 3:**
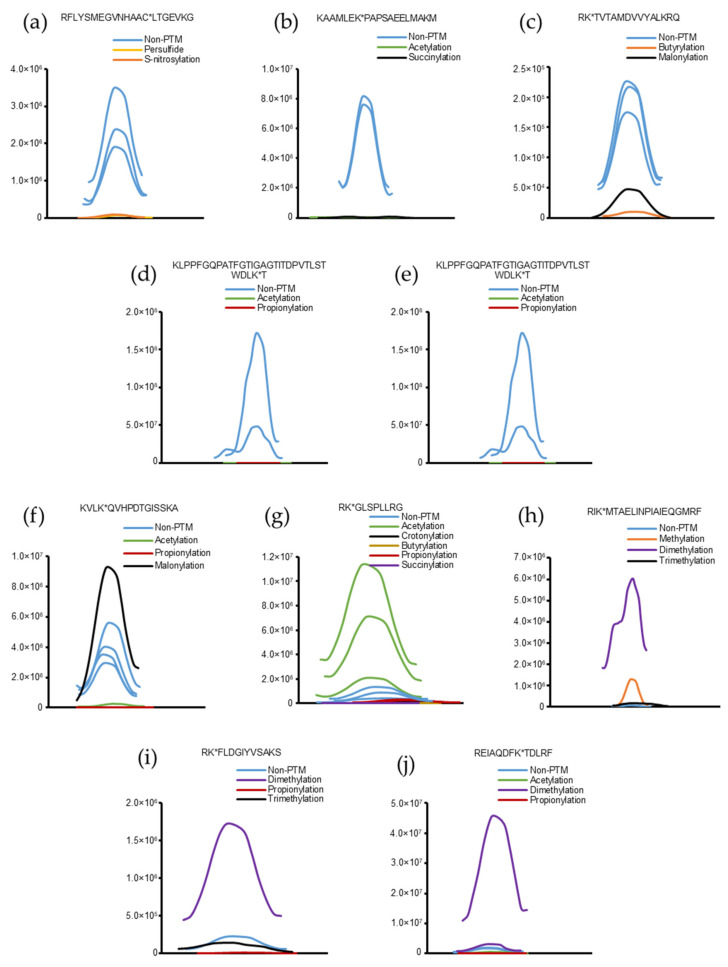
Representative peaks of intact and modified peptides. (**a**–**e**) Intact peptides have higher intensities than modified peptides. (**f**–**j**) Modified peptides have higher intensities than intact peptides.

Some peptides were chosen from Table S4 to evaluate the effects of the PTM search on peptide quantification. The base peak chromatograms of the corresponding intact and modified peptides are shown in Figure 3. It is evident that, in some cases, the intensities of the intact peptides are superior to those of the modified peptides (Figure 3a–e). However, in some instances, the intensities of the modified peptides were higher than those of the intact peptides (Figure 3f–j).

To elucidate the effects of PTM search on peptide and protein quantification, fractions E1–E5 were subjected to MaxQuant analysis. Methylation and acetylation were selected for generating the quantitative data. The intensities of peptides in the non-PTM and PTM data are listed in Table S5. Among the 427 peptides that were found to contain PTMs (methylation and acetylation), 336 were newly identified in the PTM search. The intensities of 91 other modified peptides were unchanged or increased. In the PTM search, the intensities of peptides represent the total intensities of intact peptides and modified peptides of the same sequence. Thus, the ratio of peptide intensities between the PTM search and non-PTM search indicated the relative intensities between modified and intact peptides. A ratio of 1 (7 peptides) indicated that the modified peptides had intensities of ~0. A ratio of 1.01–1.12 (60 peptides) suggested that the modified peptides had negligible intensities (≤12%) compared with those of the intact peptides. A ratio of 1.20–1.74 (10 peptides) indicated that the modified peptides had low intensities (20–74%) compared with those of the intact peptides. A ratio of 2–14.21 (13 peptides) suggested that the modified peptides had equal or higher intensities than those of the intact peptides. These findings support the results above, which manually defined base peak chromatograms of peptides.

As presented in Table S6, 401 proteins were found to have PTMs (acetylation and/or methylation). Among them, 193 proteins were newly identified and quantified after performing the PTM search. The ratio of protein intensities between PTM and non-PTM datasets reflected the effects of PTM search on protein quantification. Briefly, six proteins suffered reduced intensities, and 18 proteins showed unchanged intensities. In addition, the intensities of 103 proteins increased slightly (ratios of 1–1.2), and intensities of 34 proteins increased substantially (ratios of 1.2–2.0). Notably, 34 proteins exhibited 2–10-fold increases, and 13 proteins showed dramatic increases (>10 fold) in their intensities. Remarkably, the intensity of protein R1D656 (uncharacterized protein) increased 390.19 times after the PTM search. This was due to the substantial contribution of a methylated peptide (SLAHGSQPGQQQGVRGKGDGK) with an intensity of 5.37 × 10^9^, whereas the intensity of protein in the non-PTM search was only 1.38 × 10^7^.

Thus, the quantification of proteins changed after the incorporation of PTM search because of the variation in peptide quantification. The abundances of modified peptides might be minor or predominant compared with those of the intact peptides (Figure 3). Overall, the protein intensities increased after executing the PTM search. Protein quantification in bottom-up proteomics can provide the intensities of protein groups in the samples. With the incorporation of PTM search, protein intensity is the overall intensity of its intact and modified peptides, that is, the total intensity of its different proteoforms. Supposing that the biological functions of the proteoforms of the same family are similar, these findings suggest that conventional quantitative analysis of peptides and proteins may be biased due to the lack of modified peptide quantification. However, the biological functions of different proteoforms in the same proteoform family can vary considerably [38]. In these cases, bottom-up proteomics cannot be used to identify and quantify proteoforms. Top-down proteomics is possibly a more suitable approach to overcome this limitation [39].

### 2.4. Biological Relevance of Modified Proteins of Emiliania huxleyi

**Figure 4:**
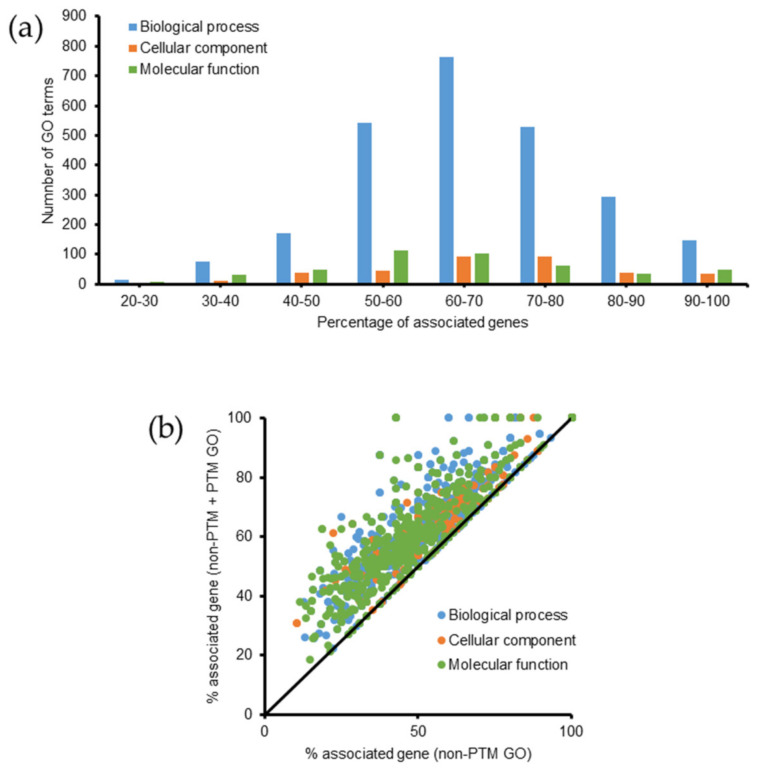
Summary of gene ontology analysis in *Emiliania huxleyi* using ClueGO. Lists of proteins from the non-PTM and PTM datasets were used for the analysis. (**a**) Summary of the number of GO terms according to the percentage of associated genes. (**b**) Increase in the percentage of associated genes after performing PTM search.

A list of identified proteins (non-PTM + PTM datasets) were subjected to gene ontology (GO) analysis using ClueGO via Cytoscape. The database of *Emiliania huxleyi* (updated on 31 October 2019) consisted of 2802 biological processes (9885 genes), 509 cellular components (8596 genes), and 1452 molecular functions (12,470 genes). Overall, 3336 GO terms, including 2532 biological processes, 354 cellular components, and 450 molecular functions were identified. The percentages of genes associated with these GO terms are shown in Figure 4a. Approximately 81% of GO terms had 50–90% associated genes. We compared GO terms according to the percentage of associated genes between the two datasets. After adding the list of modified proteins, the percentage of associated genes increased or remained unchanged for all GO terms (Figure 4b). Table S7 lists all the GO terms with a *p*-value ≤ 0.05, including 276 biological processes, 81 cellular components, and 127 molecular functions, and shows the differences between the two datasets (PTM + non-PTM versus non-PTM) regarding the percentage of associated genes. Compared with the previous GO data, some new GO terms were identified with a *p*-value ≤ 0.05, including 47 biological processes (e.g., 3-hydroxyacyl-CoA dehydrogenase activity, DNA packaging, cellular lipid metabolic process, oxidoreduction coenzyme metabolic process, and protein metabolic process), 1 cellular component (respirasome), and 17 molecular functions (e.g., transferase activity (transferring acyl groups, acyl groups converted into alkyl on transfer), 3-hydroxyacyl-CoA dehydrogenase activity, oxidoreductase activity acting on the CH-NH_2_ group of donors, protein-Lys *N*-methyltransferase activity, and Lys *N*-methyltransferase activity).

**Figure 5:**
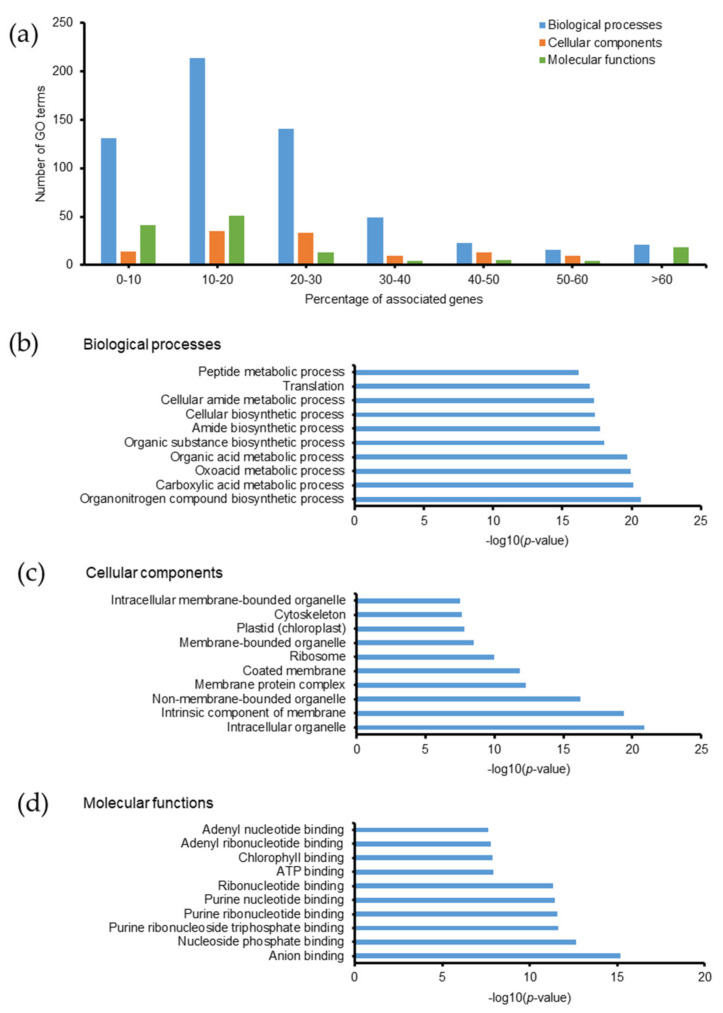
Gene ontology of methylated proteins in *Emiliania huxleyi* using ClueGO. (**a**) Summary of the number of GO terms according to percentage associated genes. Top 10 (**b**) biological processes, (**c**) cellular components, and (**d**) molecular functions based on −log(*p*-value).

PTMs of proteins usually relate to many biological functions, such as modulation of protein activity, stability, and subcellular localization by revealing or concealing active sites and altering their three-dimensional structures [40,41]. Some PTMs, including phosphorylation, acetylation, and ubiquitination, are involved in protein-protein interactions [42]. In this study, we examined the biological relevance of methylated, persulfidated, and phosphorylated proteins using GO analysis and the Kyoto Encyclopedia of Gene and Genomes (KEGG) pathway. The current database of *Emiliania huxleyi* consisted of 104 KEGG pathways (3259 genes). Methylated proteins related to 845 GO terms, most of which exhibited ≤30% associated genes (Figure 5a). Among them, 340 biological processes, 87 cellular components, 81 molecular functions, and 21 KEGG pathways were identified with a *p*-value ≤ 0.05 (Tables S8A and S9). The top 10 GO terms with the highest −log_10_(*p*-value) are listed in Figure 5b–d. Methylated proteins are involved in various metabolic and biosynthetic processes, located in different parts of the cell (intracellular organelles, cytoskeleton, plastid, ribosome, and membranes), and perform a number of functions, such as binding and enzymatic catalysis. Protein methylation is the transfer of methyl groups from S-adenosyl methionine to proteins catalyzed by methyltransferases [43]. Protein methylation has been widely studied in histones, and methylated histones can epigenetically repress or activate gene expression depending on the position of the methylated residues [44]. In *Emiliania huxleyi*, we observed methylation of Glu55 and Glu60 in histone H2A, Glu30 in histone H2B, and Glu136 in histone H4. Methylation also occurred on Glu106 in histone acetyltransferase and Cys316 in histone deacetylase. There are still hurdles in understanding the regulation of gene expression in *Emiliania huxleyi,* particularly during calcification. Unveiling the protein methylation profiles is essential for understanding the intricate intracellular molecular mechanisms of the coccolithophorid alga.

**Figure 6:**
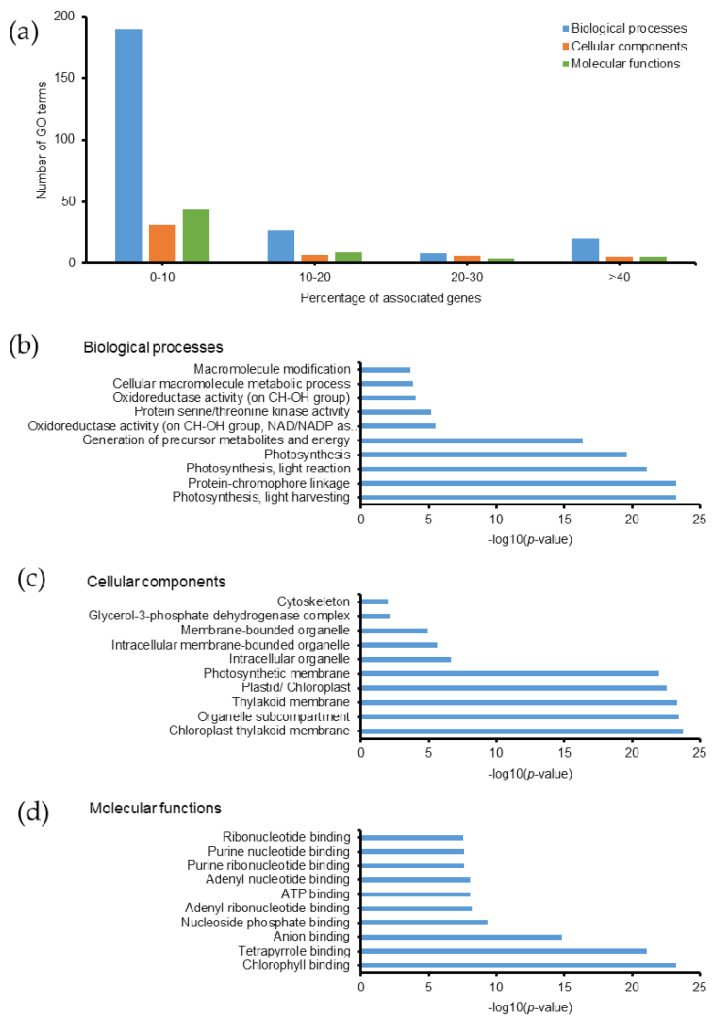
Gene ontology of persulfidated proteins in *Emiliania huxleyi* using ClueGO. (**a**) Summary of the number of GO terms according to percentage associated genes. Top 10 (**b**) biological processes, (**c**) cellular components, and (**d**) molecular functions based on −log(*p*-value).

Persulfidated proteins related to 355 GO terms, most of which exhibited ≤10% associated genes (Figure 6a). Among them, 94 biological processes, 17 cellular components, 35 molecular functions, and 6 KEGG pathways were identified with a *p*-value ≤ 0.05 (Tables S8B and S9). The top 10 GO terms with highest −log_10_(*p*-value) are listed in Figure 6b–d. Persulfidation is a potential redox mechanism that controls protein functions and various physiological processes in hydrogen sulfide (H_2_S) signaling. Understanding protein persulfidation in *Emiliania huxleyi* will provide a broad molecular basis to understand its H_2_S signaling, which is yet to be analyzed in this organism. The proteins involved in photosynthesis were persulfidated, as shown in Table S8B, which had the highest −log_10_(*p*-value) among the persulfidated proteins analyzed in the present study. These findings are in agreement with a previous study on *Arabidopsis* plants [45]. Additionally, it was reported that protein persulfidation changes enzymatic structures and activities, such as ascorbate peroxidase, glyceraldehyde-3-phosphate dehydrogenase, and glutamine synthetase [45]. In our study, we also observed persulfidation of the glycerol-3-phosphate dehydrogenase (GPDH) complex (3 proteins: R1DYC7, R1DG23, and R1BJ45, corresponding to 25% of the total number of proteins in this complex). The identification of GPDH persulfidation will enhance our knowledge of protein function in carbohydrate and lipid metabolism in coccolithophorid alga. In addition, persulfidation was detected on Asp60, Asp88, and Asp343 in glutamine synthetase (R1DWQ0), which plays a key role in nitrogen metabolism and thus, will help understand the details of the related mechanisms and protein functions in *Emiliania huxleyi*. Another study found that the majority of persulfidated proteins were located in the cytosol and chloroplasts [46]. Our data showed that 37 proteins, corresponding to 27.4% of the total number of proteins in the chloroplast, were persulfidated (GO:0009507). In addition, hydrogen sulfide enhances photosynthesis in *Spinacia oleracea* seedlings by facilitating chloroplast biogenesis, photosynthetic enzyme expression, and thiol redox modification [47]. In our study, 35 proteins located in the chloroplast thylakoid membrane were persulfidated, corresponding to 31.8% of the total number of proteins in the chloroplast thylakoid membrane (Table S8, GO:0009507).

**Figure 7:**
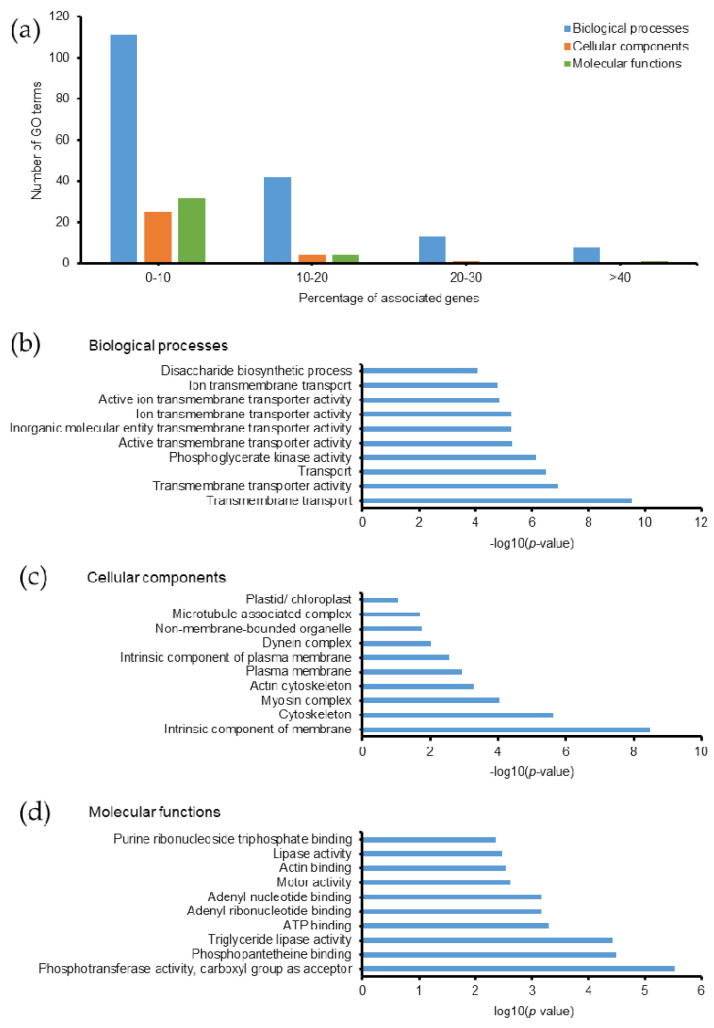
Gene ontology of phosphoylated proteins in *Emiliania huxleyi* using ClueGO. (**a**) Summary of the number of GO terms according to percentage associated genes. Top 10 (**b**) biological processes, (**c**) cellular components, and (**d**) molecular functions based on −log(*p*-value).

Protein phosphorylation is one of the most important PTMs in eukaryotic cells, which commonly occurs on tyrosine (Tyr, Y), serine (Ser, S), and threonine (Thr, T) residues. It is a reversible addition of a phosphate group catalyzed by protein kinases. Phosphorylation regulates cellular metabolism, enzymatic reactions, protein-protein interactions, and protein degradation [48]. In eukaryotes, phosphorylation of proteins is considered a key regulatory mechanism in some biological processes, such as acclimation of photosynthesis to the environment [49]. In this study, we found that phosphorylated proteins related to 243 GO terms; most of them had ≤20% associated genes (Figure 7a). Among them, 101 biological processes, 12 cellular components, 19 molecular functions, and 5 KEGG pathways were identified with a *p*-value ≤ 0.05 (Tables S8C and S9). The top 10 GO terms with the highest −log_10_(*p*-value) are listed in Figure 7b–d. In particular, proteins involved in the transport processes of *Emiliania huxleyi* are the prominent GO terms with the highest −log_10_(*p*-value). Regulation of transporter activity is crucial during the calcification process in the coccolithophorid alga. Various intracellular signaling pathways are also controlled by phosphorylation in eukaryotic cells [50]. Unfortunately, in the absence of enrichment methods, the number of phosphorylated sites and proteins in our study were 1120 and 789, respectively, which are relatively lower than those in recent phosphoproteomic studies [51]. Thus, the biological relevance of phosphorylated proteins in *Emiliania huxleyi* needs to be examined together with a phosphoproteomic analysis performed using enrichment methods to specifically understand protein phosphorylation in this organism.

The global discovery of PTMs using Comet search on a 3D-LC separation dataset is a quick and straightforward approach and can identify various types of PTMs simultaneously. The in-depth proteome coverage dataset increased the identification of intact and modified peptides. It is a useful strategy to apply to species whose PTMs have not yet been studied, such as *Emiliania huxleyi*. The first draft of the *Emiliania huxleyi* PTMs presented in this study may provide a useful initial framework for future research. The limitation of this approach is the lack of in-depth discovery of some PTMs, such as phosphorylation.

## 3. Materials and Methods

### 3.1. Data Set

A data set of *Emiliania huxleyi* cell lysates consisting of 70 peptide fractions was used for identification of PTMs. Sample preparation and MS analysis have been previously reported [27]. The raw files are available on the ProteomeXchange Consortium via the PRIDE partner repository [52], under the dataset identifier PXD018511. Data analyses were performed using the TPP version 5.1.0 [9]. The raw data files were converted to mzXML format using MSConvert [53]. Peak list files were searched against a database of *Emiliania huxleyi* (CCMP371) obtained from Uniprot with 35,707 protein entries using Comet (version 2017.01 rev.1) [8].

### 3.2. Non-PTM and PTM Search

Peptide search without PTM identification (non-PTM search) was previously performed using TPP version 5.1.0 [27], and the non-PTM data were reused in this study. PTM identification was also performed using TPP with certain changes to the search parameters. The MS/MS spectra were searched individually with different variable modifications as follows: (1) acetylation of Lys (+42.0106 Da) and 2-hydroxy isobutyrylation of Lys (+86.0368 Da); (2) ADP ribose addition of Cys/Asp/Lys/Arg (+541.0611 Da); (3) beta-methylthiolation of Asp (+45.9877 Da) and biotinylation of Lys (+226.0776 Da); (4) butyrylation of Lys (+70.0419 Da) and crotonylation of Lys (+68.0262 Da); (5) dimethylation of Lys/Arg (+28.0313 Da) and diphthamide of His (+142.1106 Da); (6) farnesylation of Cys (+204.1878 Da) and geranylation of Cys (+272.2504 Da); (7) hydroxyfarnesylation of Cys (+220.1827 Da) and S-nitrosylation of Cys (+28.9902 Da); (8) hydroxymethylation of Asn (+30.0106 Da) and hydroxytrimethylation of Lys (+59.0470 Da); (9) malonylation of Lys: +86.0004 Da and propionylation of Lys (+56.0262 Da); (10) methylation of Cys/Glu/Gln/Lys/Arg (+14.0157 Da); (11) myristoylation of Cys/Lys (+210.1984) and persulfidation of Cys/Asp (+31.9721 Da); (12) oxidation to nitro of Trp/Tyr (+44.9851 Da); (13) palmitoylation of Cys/Lys/Ser/Thr (+238.2297 Da); (14) phosphorylation of Ser/Thr/Tyr (+79.9663 Da); and (15) succinylation of Lys (+100.0160 Da) and trimethylation of Lys (+42.0470 Da). Static carbamidomethylation of Cys and variable modifications (Met oxidation and carbamylation of protein in N-term) were kept for all PTM searches [54]. For PTMs related to Cys modification, the static carbamidomethylation of Cys was changed to a variable modification. The search results were analyzed using PeptideProphet [55], and the FDR was set at 0.01.

The data were processed and visualized using Microsoft Excel 2016. Modified peptides were extracted from each search and compiled into a final PTM dataset. Peptides with similar backbones and modified sites (manually validated) were regarded as duplicates when counting the number of modified sites. Multiple modified sites were also manually curated, and representative peaks were manually extracted from the raw files. GO [56] and KEGG pathway [57] were categorized using Cytoscape version 3.7.1 (National Institute of General Medical Sciences, Bethesda, MD, USA) via ClueGO version 2.5.4 (Cordeliers Research Center, Paris, France) with a *p*-value ≤ 0.05. All modified proteins, methylated proteins, persulfidated proteins, and phosphorylated proteins were subjected to GO analysis.

### 3.3. Peptide and Protein Quantification

Peptide and protein quantification was performed using MaxQuant version 1.5.8.3 to investigate the effects of the PTM search on the intensity of proteins. Five raw files of fractions E1–E5 were searched with a built-in Andromeda search engine against the database. The parameters were set as follows: maximum two missed cleavages with trypsin, carbamidomethylation of Cys (+57.0215 Da) as a fixed modification, oxidation of Met (+15.995 Da), carbamylation at N-term (+43.0006 Da), acetylation of Lys (+42.0106 Da), and methylation of Glu/Lys/Arg (+14.0157 Da) as variable modifications, 20 ppm for first search peptide tolerance, 4.5 ppm for main search peptide tolerance, and FDR cutoff of 1%.

## 4. Conclusions

In this study, global PTM discovery was carried out without enrichment for particular types of PTMs. The global PTM search revealed a wide variety of PTMs in deep proteomic datasets. More than 13,400 PTMs were identified in 7421 proteins. Following the PTM search, the total number of peptides and protein identification increased considerably. The PTM search also affected protein quantification. GO analysis suggested that persulfidation occurred in many photosynthesis-related proteins and phosphorylation occurred in various membrane proteins in *Emiliania huxleyi*. The combination of a 3D-LC separation method with a global PTM search is a promising approach for the global discovery of PTMs in *Emiliania huxleyi.* This strategy can be further applied to other species to provide an overview of their PTMs and facilitate studies on specific PTMs.

## Data Availability

Publicly available raw MS/MS files were analyzed in this study. This data can be found here: https://www.ebi.ac.uk/pride/archive?keyword=PXD018511, accessed on 13 October 2020.

## Supplementary Materials

The following are available online, Table S1: List of modified peptides in *Emiliania huxleyi* (some peptides have two different types of PTMs and are listed twice), Table S2: Distribution of PTMs by PTM types and modified amino acid sites, Table S3: Summary of PTM type and site in each protein, Table S4: List of multiply modified sites, Table S5: Comparison of peptide intensities between non-PTM and PTM search using MaxQuant (only modified peptides are listed), Table S6: Comparison of protein intensities between non-PTM and PTM search using MaxQuant (only modified proteins are listed), Table S7: Comparison of gene ontology before and after addition of modified proteins: (A) biological processes, (B) cellular components, and (C) molecular functions, Table S8: Gene ontology of (A) methylated, (B) persulfidated, and (C) phosphorylated proteins of *Emiliania huxleyi* CCMP371; and (D) list of protein IDs used for gene ontology analysis with Cytoscape, Table S9: KEGG pathways of methylated, persulfidated, and phosphorylated proteins of *Emiliania huxleyi* CCMP371.

## Sample Availability

Samples of *Emiliania huxleyi* are available from the authors.
